# Monocyte Dysfunction, Activation, and Inflammation After Long-Term Antiretroviral Therapy in an African Cohort

**DOI:** 10.1093/infdis/jiz320

**Published:** 2019-07-19

**Authors:** Rose Nabatanzi, Lois Bayigga, Stephen Cose, Sarah Rowland Jones, Moses Joloba, Glenda Canderan, Damalie Nakanjako

**Affiliations:** 1 Department of Immunology and Molecular Biology, Makerere University College of Health Sciences, Kampala, Uganda; 2 Medical Research Council/Uganda Virus Research Institute, Uganda Research Unit on AIDS, Entebbe, Uganda; 3 Nuffield Department of Medicine, University of Oxford, United Kingdom; 4 Department of Medicine, Makerere University College of Health Sciences, Kampala, Uganda; 5 Infectious Diseases Institute, School of Medicine, Makerere University College of Health Sciences, Kampala, Uganda; 6 Department of Pathology, Case Western Reserve University, Cleveland, Ohio

**Keywords:** HIV infection, immune activation, immune responses, long-term antiretroviral therapy, sub-Saharan Africa

## Abstract

**Background:**

Monocyte dysfunction may persist during antiretroviral therapy (ART).

**Methods:**

Frozen peripheral blood mononuclear cells of 30 human immunodeficiency virus (HIV)-infected ART-treated adults with sustained viral suppression and CD4 counts ≥500 cells/µL were consecutively analyzed for monocyte phenotypes and function.

**Results:**

Nonclassical monocytes (CD14^+^, CD16^++^), interleukin (IL)-1β production, and expression of CD40 and CD86 were lower among ART-treated HIV-infected adults relative to age-matched HIV-negative adults (*P* = .01, *P* = .01, and *P* = .02, respectively). Intestinal fatty acid-binding protein, IL6, and soluble CD14 were higher among HIV-infected adults relative to HIV-negative adults (*P* = .0002, *P* = .04, and *P* = .0017, respectively).

**Conclusions:**

Further investigation is required to understand drivers of persistent monocyte activation and dysfunction.

## Teaser

Monocyte activation with low expression of costimulatory ligands and IL-1β production were observed despite 7 years of suppressive ART. Immune activation, inflammation, and gut mucosal damage remained high, although there was no evidence of microbial translocation or cytomegalovirus infection.

Access to antiretroviral therapy (ART) has increased steadily worldwide. Approximately 21.7 million people were receiving ART by 2017, 60% of whom live in sub-Saharan Africa (SSA) [1]. Although ART improves innate and adaptive immune responses, our studies reported persistent defects in T-cell function [2]. Persistent monocyte dysfunction may affect the quality of host adaptive immune responses to pathogens. Blood monocytes, a subset of cells of the innate immune system, represent 10% of circulating leukocytes in humans. Monocytes are classified into the following: (1) classical monocytes (CD14^++^CD16^−^ monocytes) [3] that are predominantly phagocytic and exhibit high peroxidase activity in response to lipopolysaccharide (LPS); (2) nonclassical monocytes (CD14^+^CD16^++^ monocyte) that remove damaged cells and debris from circulation and are associated with wound healing and resolution of inflammation [4]; and (3) intermediate monocytes (CD14^++^CD16^+^ monocytes) that are inflammatory and produce interleukin (IL)-1β and tumor necrosis factor (TNF) in response to LPS [5]. Monocyte activation is associated with spontaneous release of proinflammatory cytokines including TNF, IL-1β and IL-6, elevated plasma levels of soluble CD14 (sCD14), and D-dimers, all of which demonstrate immune activation and inflammation in human immunodeficiency virus (HIV)-infected persons [6]. Antiretroviral therapy (ART) controls HIV replication and reduces microbial translocation and immune activation; however, it is still unclear whether these parameters return to levels comparable with healthy HIV-uninfected individuals.

Microbial translocation has been described as a major driver of immune activation in American cohorts, although its role in African populations is still controversial. In a Ugandan cohort of ART-naive adults with opportunistic infections, microbial translocation was not associated with HIV disease pathogenesis and inflammation [7]. In a South African cohort, microbial translocation and acute opportunistic infections were major drivers of monocyte activation during the first year of ART [8]. The role of monocyte activation, microbial translocation, and inflammation after several years of suppressive ART without opportunistic infections is yet to be understood. Given the potential role of monocyte activation in HIV-related cardiovascular disease risk, including atherogenesis and coronary artery calcium [9], there is a need to understand monocyte activation, its drivers, and its role during long-term ART in Africa. Moreover, monocyte activation has also been associated with accelerated immune aging among ART-treated adults. Therefore, we examined monocyte phenotypes, activation markers, and functions among HIV-infected adults with seven years of suppressive ART and CD4 T-cell count restoration to ≥500 cells/μL. We also measured serum biomarkers of immune activation and inflammation, as well as their potential drivers including gut mucosal damage, microbial translocation, inflammation, and subclinical cytomegalovirus (CMV) infection.

## METHODS

### Ethics Approval and Consent to Participate

Ethical approval was sought from the School of Biomedical Sciences Makerere University College of Health Sciences Research and Ethics Committee. All participants provided written informed consent for storage and future use of their samples in studies to understand host immune recovery during ART.

### Study Design

A comparative sample-based, cross-sectional study was conducted within the Infectious Diseases Institute HIV treatment research cohort. In 2004–2005, a cohort of 559 adults ≥18 years (386 [69%] female) with a median age of 36 (interquartile range [IQR], 21–44) years and a median CD4 count of 98 (IQR, 21–163) cells/μL initiated ART. Viral load and CD4 counts were estimated every six months. All patients received cotrimoxazole (or dapsone) prophylaxis. Adherence to ART was encouraged by at least three individual counseling sessions. Patients had monthly physician reviews to monitor adherence to medication, toxicities, and acute infections.

### Study Participants

After seven years of ART, 121 adults who remained on first-line therapy with undetectable viral loads (<50 copies) from the first measurement at six months post-ART and without opportunistic infections in the preceding six months were described by CD4 count increases from the nadir CD4 count. All the (30 [26/87%] female and median age 40 [IQR, 38–46] years) patients in the highest quartile of CD4 recovery (581–1572 cells/µL), normal CD4 counts for healthy HIV-uninfected Ugandans, were included. Stored peripheral blood mononuclear cells (PBMCs) and plasma were used. All parameters were compared with 30 age-matched, healthy, HIV-negative adults (±5 years) from the same communities. Healthy adults were selected randomly as they attended nonillness visits for health education and voluntary HIV testing. Sixty-three percent (19 of 30) were female and the median age was 35.5 (IQR 32.5–42.0) years (Supplemental Table 1).

### Experimental Procedures

#### Flow Cytometry Assays

Cryopreserved PBMCs were snap-thawed and washed with R20 (complete Roswell Park Memorial Institute [RPMI] 1640 medium supplemented with 20% fetal bovine serum [FBS]). The cells were counted and rested for 4 hours at 37°C in a 5% CO_2_ incubator. Eighty-five percent average viability was achieved after resting.

#### Surface Staining

Cells were stained with CD3 BV510, CD56 BV510, CD19 BV510, CD14 PerCP Cy5.5, CD16 BV605, CD86 APC, CD40 APC-H7, and HLAR-DR APC-R700 (BD Biosciences). Surface staining was done at 4°C for 30 minutes with saturating concentrations of antibodies in the presence of fixable live/dead stain (Invitrogen). Cells were fixed and acquired using the LSRII flow cytometer (FACSDiva software 8.0; BD Bioscience). At least 500 000 events of lymphocytes were acquired. CD3 expression was used to exclude T cells, CD19 excluded B cells, and CD56 excluded natural killer cells. Monocyte subsets were described by intensity of expression of CD14 and CD16 markers [5]. CD14^++^CD16^−^ cells were denoted classical monocytes, CD14^++^CD16^+^ were denoted intermediate monocytes, and CD14^+^CD16^++^ were denoted nonclassical monocytes (Supplemental Figure 1).

Intracellular cytokine staining was done after stimulation of 2 × 10^6^ PBMCs with 1 ng/mL LPS at 37°C in a 5% CO_2_ incubator for 4 hours in the presence of 1:250 ng/mL brefeldin A. Lipopolysaccharide stimulation was optimized at 1 ng/mL after trials with 0.5, 1, and 2 ng/mL. Cells were washed with FACS buffer (5% FBS, 0.01% sodium azide, and 1× phosphate-buffered saline. Cells were then stained with TNF PE-CY7, IL-1β PE, and IL-6 BV421 (BD Biosciences). Samples were acquired on an LSRII using BD FACSDiva software 8.0. At least 100 000 events of the monocyte gate were acquired (Supplemental Figure 1).

#### Enzyme-Linked Immunosorbent Assays 

Cryopreserved plasma was thawed and analyzed for sCD14 (catalog no. DC140), intestinal fatty acid-binding protein ([IFAB-P] catalog no. DY3078), LPS binding protein ([LBP] catalog no. KA0448), and IL-6 (catalog no. D6050) (all from R&D Systems, Abingdon, UK). We also determined circulating D-dimer levels (reference no. EHDDIMER; Thermo Fisher Scientific), LPS (catalog no. MBS2703401; MyBioSource, Inc.), and CMV antibodies (catalog no. abx354612; Abbexa Cambridge, UK). RealStar CMV PCR Kit (Altona Diagnostic, Hamburg, Germany) was used for CMV viral load.

#### Data Management and Analysis

Flow data was analyzed using FlowJo software (version 10.1; Tree Star). Comparisons and graphs were made using Graph Prism 6 and tested using Mann-Whitney *U* test for non-parametric tests with statistical significance at *P* ≤ .05.

## RESULTS

Clinical and demographic characteristics were similar among the ART-treated adults (median age 40 [IQR, 38–46] years and 26 [87%] female) and age-matched healthy HIV-negative adults (median age 35.5 [IQR, 32.5–42] years and 19 [63%] female) (Supplemental Table 1).

### Monocyte Phenotype and Function

**Figure 1.**
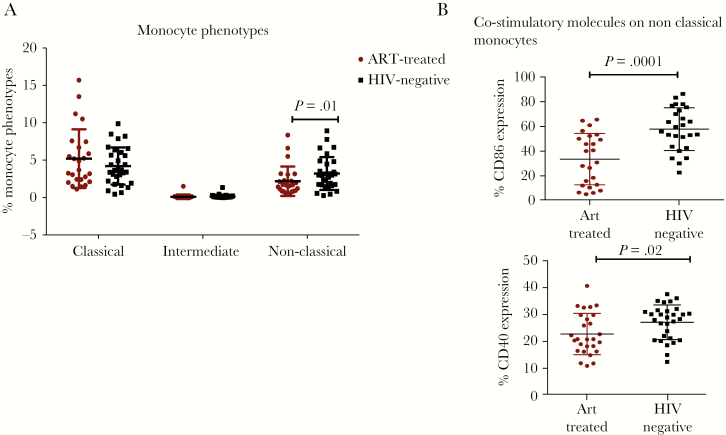
Monocyte phenotype distribution in peripheral blood and expression of costimulatory molecules (CD86 and CD40) nonclassical monocytes. (A) Monocyte phenotypes among the antiretroviral therapy (ART)-treated human immunodeficiency virus (HIV)^+^ individuals with CD4 count of >500 cell/µL and supressed viral load in comparison with the age-matched, healthy, HIV-negative population from the same community. (B) CD86 and CD40 expression on nonclassical monocytes of ART-treated HIV-infected and HIV-negative individuals after 1 ng/mL lipopolysaccharide stimulation. The Mann-Whitney *U* test was used to compare the monocyte proportions between HIV-infected and healthy subjects.

**Figure 2.**
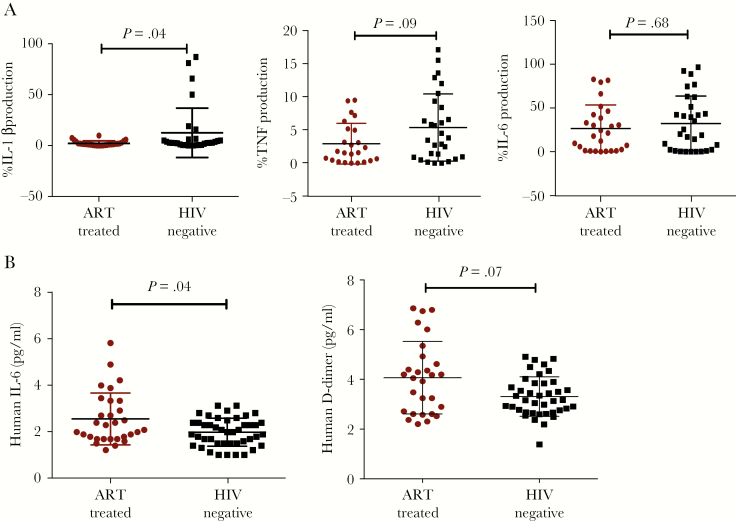
Interleulin (IL)-1β production by the classical monocytes and circulating inflammation markers IL-6 and D-dimer. (A) A comparison of IL-1β production among antiretroviral therapy (ART)-treated human immunodeficiency virus (HIV)-infected and healthy HIV-negative individuals from the same community. (B) Circulating IL-6 and D-dimer among ART-treated HIV-infected and HIV-negative individuals. The Mann-Whitney *U* test was used to compare parameters between HIV-infected and healthy subjects, with statistical significance at *P* < .05.

Proportions of nonclassical monocytes (CD14^+^, CD16^++^) were lower in the ART-treated HIV-infected individuals than in their HIV-negative counterparts (*P* = .01); classical (CD14^++^, CD16^−^) and intermediate monocyte (CD14^++^CD16^+^) subsets were similar (Figure 1A). CD86 and CD40 expression by nonclassical monocytes after 4-hour LPS stimulation was lower among HIV-infected than HIV-negative individuals (*P* = .0001 and *P* = .02, respectively) (Figure 1B). We observed lower IL-1β production by classical monocytes among ART-treated HIV-positive relative to healthy HIV-negative adults (*P* = .04) (Figure 2A).

### Monocyte Activation and Inflammation

Serum IL-6 was higher among ART-treated HIV-infected than HIV-negative individuals (*P* = .04), and serum levels of D-dimers were comparable in both groups (Figure 2B). Circulating sCD14 was higher among ART-treated HIV-infected (median 12754.2 [IQR, 8368.3–14 940.8] pg/mL) than among healthy HIV-negative individuals (median 9010 [IQR, 5549.0–12298.7] pg/mL; *P* = .002) (Supplemental Table 2).

### Drivers of Activation and Inflammation

Soluble I-FAB-P was higher among ART-treated HIV-infected than HIV-negative individuals (*P* = .0002). Serum levels of LBP and LPS were lower among ART-treated HIV-infected than HIV-negative individuals (*P* = .0018) and (*P* = .0006), respectively. Serum CMV viremia and immunoglobulin G levels were comparable among ART-treated HIV-infected relative to HIV-negative individuals (Supplemental Table 2).

## DISCUSSION

### Monocyte Phenotypes and Function

Low circulating nonclassical monocytes among ART-treated adults is likely due to continuous death of this phenotype, which is more permissive to HIV infection due to its high expression of CCR5. The nonclassical monocytes have high expression of CD16^+^ and FcγRIII and are known to have a unique ability of patrolling resting vasculature to remove debris and repair damaged tissue [10]. The low nonclassical monocytes exhibited by ART-treated HIV-infected adults without coinfections may play a role in the noncommunicable disease risk associated with chronic HIV disease [11].

Low interleukin-1β (IL-1β) production by classical monocytes of HIV-infected individuals demonstrated impaired monocyte immune responses to invading antigens. Interleukin-1β contributes to the control of bacterial, viral, parasitic, and fungal infections and has been associated with protection against tuberculosis [12]. Oversecretion of 1L-1β, IL-6, and TNF-α in ART-naive patients was observed in a French cohort (MANIF 2000 Study Group), which reduced during ART. Although cytokine production had greatly decreased, the cells still produced good amounts of cytokines upon in vitro LPS stimulation. Lipopolysaccharide did not stimulate significant production of IL-1β by monocytes in our study. We postulate that exposure to infections in SSA causes immune activation with subsequent anergy.

Co-stimulatory molecules CD40 and CD86, expressed on monocytes and other antigen presenting cells, are critical in ensuring optimal antigen presentation to T-cells and effective activation. Engagement of co-stimulatory ligands (CD40L and CD86) boosts antigen-stimulated cytokine production, clonal expansion of T cells, and their differentiation into effector cells [13]. Lower expression of co-stimulatory ligands in HIV-infected individuals may be associated with low T-cell effector functions. Stability of the antigen presentation process during HIV infection has been described based on major histocompatibility complex (MHC)-II expression [14], although expression of CD40 and CD86 had not been evaluated.

### Monocyte Activation and Inflammation

Monocyte activation correlates with impairment of monocyte function, similar to our observation of lower expression of costimulatory molecules with lower IL-1β production among ART-treated HIV-infected adults. Despite evidence of mucosal damage among ART-treated HIV-infected adults without evidence of microbial translocation, sCD14 marker of monocyte activation was high. In the absence of opportunistic infections including CMV infection, persistent monocyte activation could be attributed to replication of HIV in the reticuloendothelial system [8]. Although several studies have demonstrated reduction of circulating LPS during ART, some previous reports showed lower circulating LPS among HIV-infected individuals relative to HIV-negative controls. Moreover, Redd et al [7] suggested that the effect of microbial translocation on HIV might not be subclinical endotoxemia but rather the variable host responses to circulating microbial products. There is also evidence that healthy HIV-negative individuals in SSA may have a subclinical environmental enteropathy, which might be responsible for the microbial translocation observed in HIV-negative adults [15]. Human immunodeficiency virus disease adds an increase in crypt depth and lactulose permeation particularly in advanced disease with CD4 cells <200 cells/μL [15]. Low levels of circulating LPS after seven years of ART could be due to prolonged cotrimoxazole and recurrent antibiotic use, which modify gut microbial communities.

High levels of circulating IL-6 demonstrate persistent systemic inflammation, and dysregulated production of IL-6 plays a key role in chronic inflammation and autoimmunity. Unfortunately, persistent inflammation and activation during ART is associated with increased risk of fatal nonacquired immune deficiency syndrome illnesses among adults aging with HIV [9]. For example, circulating CD16^+^ monocytes were associated with atherogenesis and greater coronary artery calcium [9]. Our results are comparable with previous reports that immune activation decreased with duration of ART although it did not return to levels reduction of monocyte activation to levels comparable to HIV-negative individuals has been observed after 48 weeks of integrase inhibitor-based ART in the United States. More studies are needed to understand drivers and potential modulators of monocyte activation and inflammation, particularly among HIV-infected adults in SSA where the landscape of ART regimen, extent of disease at ART initiation, and endemic infectious agents are variable. As a limitation of our cross-sectional study design, we did not have longitudinal data. Furthermore, using stored samples may not capture prevailing biologic events that can only be demonstrable at physiologic conditions.

## CONCLUSIONS

Monocyte activation with impaired antigen presentation and IL-1β production were observed among ART-treated HIV-infected adults after 7 years of suppressive ART. Commonly implicated drivers of immune activation such as CMV viremia and microbial translocation were absent, hence there is a need to explore other potential drivers of monocyte activation and inflammation among aviremic ART-treated adults.

## SUPPLEMENTARY DATA

Supplementary materials are available at *The Journal of Infectious Diseases* online. Consisting of data provided by the authors to benefit the reader, the posted materials are not copyedited and are the sole responsibility of the authors, so questions or comments should be addressed to the corresponding author.

**Supplemental Table 1.** Demographic characteristics of human immunodeficiency virus (HIV)-infected adults after 7 years of suppressive antiretroviral therapy (ART) and age- and gender-matched health HIV-negative counterparts from the same community.

**Supplemental Table 2.** Comparison of levels of serum biomarkers of microbial translocation and monocyte activation between antiretroviral therapy (ART)-treated and human immunodeficiency virus (HIV)-negative individuals.

**Supplemental Figure 1.** Monocyte gating strategy. (A) shows how monocytes were gated using light scatter characteristics and the exclusion of T cells, B cells, natural killer (NK) cells, and dead cells by gating on the CD3^−^, CD19^−^, CD56^−^, and Aqua^−^ cells, respectively. HLADR^+^ gate shows HLADR^+^ monocytes and gating strategy for the different monocyte subsets. CD16^++^ the nonclassic monocytes (NC), CD14^++^, CD16^+^ intermediate monocytes (IN), and CD14^++^ classic monocytes (C). (B) shows the gating strategy for interleukin (IL)-1beta, tumor necrosis factor (TNF), and IL-6 production upon stimulation of peripheral blood mononuclear cells (PBMCs) with 1 ng/mL lipopolysaccharide (LPS).

**Supplemental Figure 2.** Gating strategy for costimulatory molecules CD86 and CD40 by nonclassic monocytes of antiretroviral therapy (ART)-treated human immunodeficiency virus (HIV)-infected and HIV-negative individuals after 1 ng/mL lipopolysaccharide (LPS) stimulation.

jiz320_suppl_Supplementary_Material

## Notes

***Acknowledgments*.** We acknowledge funding from the Alliance for Global Health and Science at the University of California, Berkeley, and the Wheeler Center for Emerging and Neglected Diseases, University of California, Berkeley, which provided funding for our research to further understand recovery of innate immune cells during antiretroviral therapy.

***Author contributions.*** R. N., L.B., G.C., S. C., M. J., S. R. J., and D. N. contributed to the conceptualization and execution of the research. R. N. and D. N. drafted the manuscript. All authors reviewed and approved the manuscript for publication.

***Disclaimer*.** The views expressed in this publication are those of the author(s) and not necessarily those of African Academy of Sciences, New Partnership for Africa’s Development Planning and Coordinating Agency, Wellcome Trust, or the United Kingdom government.

***Financial support.*** This work was funded through the DELTAS Africa Initiative (Grant Number 107743/Z/15/Z), which provided funding to D. N. and R. N. through a group leader award. The DELTAS Africa Initiative is an independent funding scheme of the African Academy of Sciences’s Alliance for Accelerating Excellence in Science in Africa and supported by the New Partnership for Africa’s Development Planning and Coordinating Agency with funding from the Wellcome Trust (Grant Number 107743/Z/15/Z) and the United Kingdom government.

***Potential conflicts of interest.*** All authors: No reported conflicts of interest. All authors have submitted the ICMJE Form for Disclosure of Potential Conflicts of Interest.
