# Supplementary material for: Monocyte Dysfunction, Activation, and Inflammation After Long-Term Antiretroviral Therapy in an African Cohort
Source: J Infect Dis. 2019 Jul 19;220(9):1414–9. doi: 10.1093/infdis/jiz320 (PMC6761975; doi:10.1093/infdis/jiz320)
Supplement: jiz320_suppl_Supplementary_Material [file jiz320_suppl_supplementary_material.pdf]

Supplemental table 1: Demographic characteristics of HIV-infected adults after seven years of suppressive ART and age-and gender matched health HIV-negative counterparts from the same community

**Characteristics¥**	**ART-treated optimal responders* N=30**	**Healthy HIV-negative N=30**
Age [median (IQR)], years	40 (38, 46)	35.5 (32.5,42)
Female gender n (%)	26 (87)	19 (63)
Baseline CD4 count: median (IQR) cells/µl	97 (11, 158)	N/A
Current CD4: median (IQR) cells/µl	607 (759, 996)	N/A
BMI; median (IQR)	22.57(20,25)	25.95(22, 30)
Hypertension (%)	2 (6.7)	2 (6.7)
Diabetes (%)	1 (3.3)	1(3.3)
Fever	0	0
**Current Regimen**		
ZDV-3TC-NVP (%)	56.7	N/A
ZDV-3TC-EFV (%)	30.0	N/A
TDF-3TC-EFV (%)	3.3	N/A

***All optimal responders started ART at CD4 counts <200 cell/µl and had sustained viral suppression from the first viral load test after six months of ART. ¥ There was no statistically significant difference in characteristics of ART-treated and healthy HIV-negative individuals.

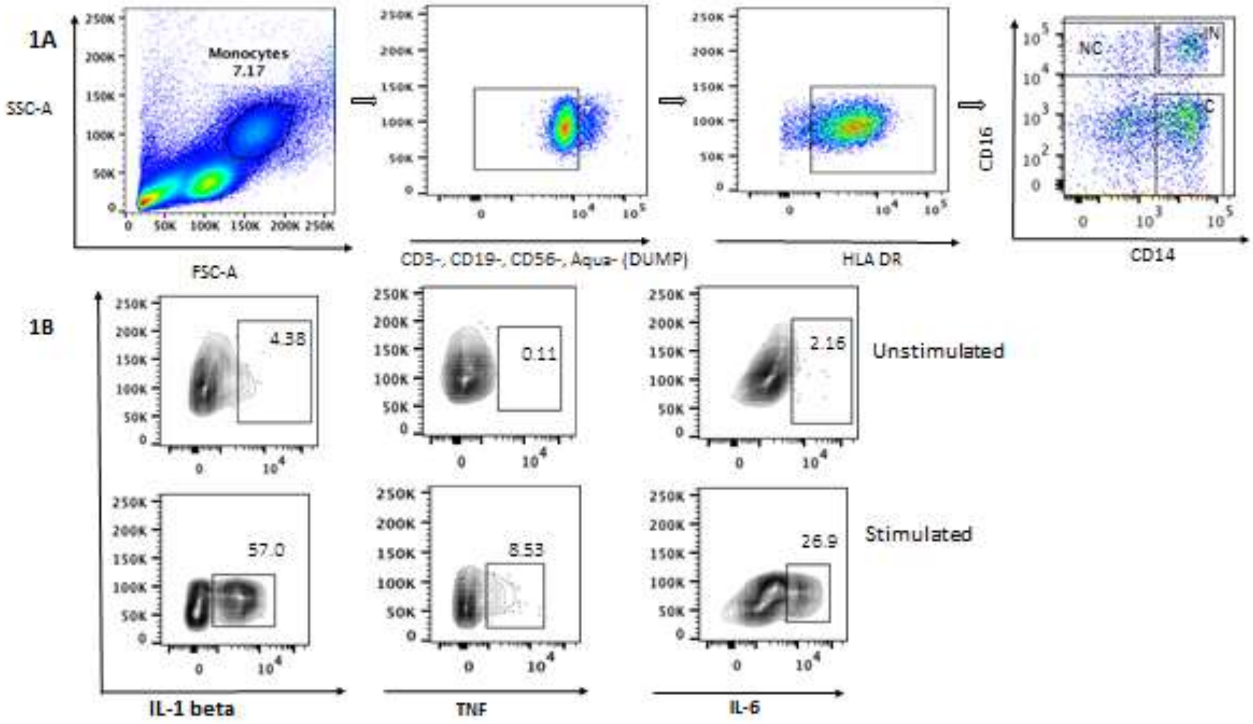

Supplemental figure 1: Monocyte gating strategy. A shows how monocytes were gated using light scatter characteristics and the exclusion of T cells, B cells, NK cells and dead cells by gating on the CD3-, CD19-, CD56- and Aqua- cells respectively. HLADR+ gate shows HLA DR+ monocytes and gating strategy for the different monocyte subsets. CD16+ the non-classical monocytes (**NC**), CD14+, CD16+ Intermediate monocytes (**IN**) and CD14+ Classical monocytes (**C**). **B** shows the gating strategy for IL-1beta, TNF and IL-6 production upon stimulation of PBMCs with 1ng/ml of LPS.

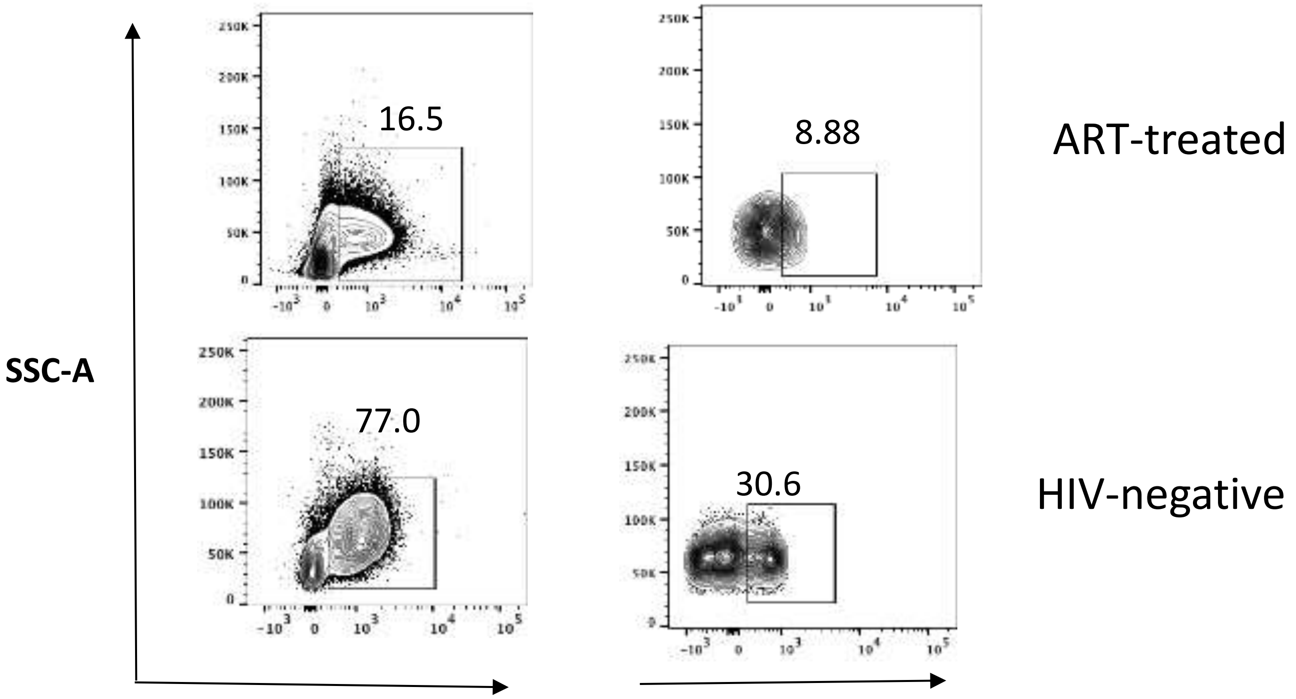

Supplemental figure 2: CD86 and CD40 receptor expression on non-classical monocytes of ART-treated HIV-infected and HIV negative individuals after 1ng/ml of lipopolysaccharide (LPS) stimulation. The Mann–Whitney *U* test was used to compare the monocyte proportions between HIV-infected and healthy subjects.

Supplemental table 2: Comparison of levels of serum biomarkers of microbial translocation and monocyte activation between antiretroviral therapy (ART)-treated and HIV negative individuals

**Microbial Translocation**	**ART-treated individuals Median (IQR) pg/ml**	**HIV negative individuals Median (IQR) pg/ml**	**P value**
I-FABP	25.87 (16.41,71.91)	16.20 (8.899,22.26)	0.0002
LPS	33.69 (25.58, 41.69)	57.04 (14.79,102.5)	0.0006
LBP	29.50 (16.37, 37.02)	41.85 (31.34, 58.20)	0.0018
**Monocyte activation**			
sCD14	12754.2 (8368.3,14940.8)	9010.39 (5549.0,12298.7)	0.0017
CMV antibodies*	1.716 (0.9745,2.149)	1.325 (0.706,1.976)	0.09

Abbreviations: Intestinal fatty acid binding protein (**IFAB-P**) – a biomarker for intestinal barrier dysfunction, Lipopolysaccharide (**LPS**)- a Glycolipids found on some membranes of gram negative bacteria, Lipopolysaccharide binding protein(**LBP**)-an acute phase protein that bind to bacterial LPS, soluble CD14(**sCD14**)-non-specific marker for monocyte activation and Cytomegalo Virus (**CMV**)- Herpes Virus.

*****CMV viremia was negative among ART-treated HIV-infected and healthy HIV-negative adults.
